# The Trypanosome Exocyst: A Conserved Structure Revealing a New Role in Endocytosis

**DOI:** 10.1371/journal.ppat.1006063

**Published:** 2017-01-23

**Authors:** Cordula M. Boehm, Samson Obado, Catarina Gadelha, Alexandra Kaupisch, Paul T. Manna, Gwyn W. Gould, Mary Munson, Brian T. Chait, Michael P. Rout, Mark C. Field

**Affiliations:** 1 Wellcome Trust Centre for Anti-Infectives Research, School of Life Sciences, University of Dundee, Dow Street, Dundee, United Kingdom; 2 The Rockefeller University, 1230 York Avenue, New York, NY, United States of America; 3 School of Life Sciences, University of Nottingham, Nottingham, United Kingdom; 4 Institute of Molecular, Cell and Systems Biology, College of Medical, Veterinary and Life Sciences, University of Glasgow, Glasgow, United Kingdom; 5 Department of Biochemistry and Molecular Pharmacology, University of Massachusetts Medical School, Worcester, MA, United States of America; University Wuerzburg, GERMANY

## Abstract

Membrane transport is an essential component of pathogenesis for most infectious organisms. In African trypanosomes, transport to and from the plasma membrane is closely coupled to immune evasion and antigenic variation. In mammals and fungi an octameric exocyst complex mediates late steps in exocytosis, but comparative genomics suggested that trypanosomes retain only six canonical subunits, implying mechanistic divergence. We directly determined the composition of the *Trypanosoma brucei* exocyst by affinity isolation and demonstrate that the parasite complex is nonameric, retaining all eight canonical subunits (albeit highly divergent at the sequence level) plus a novel essential subunit, Exo99. Exo99 and Sec15 knockdowns have remarkably similar phenotypes in terms of viability and impact on morphology and trafficking pathways. Significantly, both Sec15 and Exo99 have a clear function in endocytosis, and global proteomic analysis indicates an important role in maintaining the surface proteome. Taken together these data indicate additional exocyst functions in trypanosomes, which likely include endocytosis, recycling and control of surface composition. Knockdowns in HeLa cells suggest that the role in endocytosis is shared with metazoan cells. We conclude that, whilst the trypanosome exocyst has novel components, overall functionality appears conserved, and suggest that the unique subunit may provide therapeutic opportunities.

## Author Summary

Trafficking is an important process in most eukaryotic cells, and has been adapted for specific species to accommodate their requirements. There is good evidence for the innovation of novel routes and mechanisms in plants and animals in terms of the evolution of lineage-specific proteins that function within the endocytic and exocytic pathways, and emerging data indicates that similar novel features are also present in unicellular protozoa. This suggests the likely presence of significant variation within membrane trafficking. Here we have identified a novel component of the exocyst, a complex that is important in late stages of protein secretion. Moreover, we also find evidence that the exocyst is involved in endocytosis extending the functional reach of this complex.

## Introduction

Nutrient uptake, immune evasion, developmental progression and sensitivity to several drugs all require the action of the membrane transport system in trypanosomes [1]. In African trypanosomes, maintaining the composition and homeostasis of the surface proteome is closely connected to many of these processes, including clearance of acquired immune effectors from the surface coat, resistance to innate immune factors, uptake of heme and iron together with sensitivity to suramin and possibly additional trypanocides [2,1]. The membrane transport system of *Trypanosoma brucei* is comparatively well studied, and whilst generally conserved with other eukaryotes in overall architecture [3], the system has been adapted to the parasitic life style and the specific challenges associated with survival in the mammalian bloodstream [4,5,1]. Endocytic mechanisms have received significantly more attention than secretory pathways due in part to technical tractability, but despite the clear importance of secretory pathways for the biosynthesis of the surface coat.

The membrane trafficking system is mediated by a core of paralogous protein families, specifically Rabs, SNAREs, vesicle coats and tethering complexes. For the most part these are well conserved and play similar roles across eukaryotes [3]. The SNARE and Rab families are large, and individual members associated with distinct subcellular organelles controling transport between compartments [6–10]. In contrast, the multiple membrane-tethering complexes (MTCs) have distinct compositions from each other, suggesting mechanistic differentiation *albeit* with some evidence for common evolutionary descent through the CATCHR fold, shared by several subunits in multiple complexes [11–16]. MTCs include TRAPP I, II and III, COG, HOPS, CORVET, Dsl1, GARP and the exocyst, and mediate attachment of vesicles to target membranes and/or homotypic fusion between organelles. Significantly, MTCs are regulated by, and also seem to regulate, small GTPases and SNAREs. Significantly, none of the MTC complexes have been studied in trypanosomes and hence their functions and compositions remain uncharacterised.

The exocyst in *Saccharomyces cerevisiae* was originally described as comprising Sec3, 5, 6, 8, 10 and 15, with Sec15 binding Sec4, the yeast ortholog of Rab11 [17]. Exo70 and Exo84 were subsequently discovered and the holocomplex demonstrated to be a stable 19.5S particle [18–20]. Overall the exocyst forms a rod, and is likely monomeric, with the vast majority of proteins adopting structures that are near exclusively α-helical bundles [19,21]. Based on interactions with multiple plasma membrane GTPases [22], the exocyst is proposed to tether secretory vesicles to the plasma membrane [15, 23]. Deliberate mistargeting of exocyst subunits to the mitochondrion recruits both additional exocyst subunits to this site and secretory cargo, supporting an exocyst function in secretion [24]. Imaging studies indicate involvement in endocytic events, as exocyst mutants display altered endocytic and post-Golgi vesicle dynamics [25], further supported by the spatial proximity of endocytic and exocytic sites in several organisms, including *Drosophila melanogaster*, yeast, and trypanosomatids [25–28].

Despite a conserved basic system of Rabs and SNAREs and major coat proteins, there is mounting evidence for divergence in trafficking mechanisms across eukaryotes, with multiple examples of lineage-specific proteins or secondary losses now known [29–32]. In *T*. *brucei*, which is highly divergent from animals and fungi, endocytosis is extremely rapid, and despite being exclusively clathrin-mediated (CME), has several protein losses and gains compared to higher eukaryotes [4,5]. Previous comparative genomics studies found only six of the eight canonical exocyst subunits in trypanosomes, with Sec5 and Exo84 evading identification [12], suggesting an alternative, simplified exocyst complex in trypanosomatids with a distinct mechanism, a novel architecture or a failure to uncover highly divergent orthologs.

To directly address this question we investigated the composition and function of the trypanosome exocyst and found evidence for all eight canonical subunits, together with a novel ninth component that has a divergent architecture from the remaining eight conserved subunits. Further, we demonstrate a clear role in endocytosis, that extends between trypanosomes and mammalian cells, suggesting a general role across eukaryotes.

## Results

### The exocyst of trypanosomes is conserved

Evidence for the exocyst complex as a distinct octameric biochemical entity was provided by immunoprecipitation and velocity gradient centrifugation [17, 19, 20, 21] with no evidence for additional stable subunits. In contrast, there is good evidence for interactions with several GTPases (Sec4/Rab11, Rho1 and 3 and Cdc42), SNAREs, Sec1 and multiple cytoskeletal components, although these are weakly bound and/or transiently interacting [22, 33–41]. An earlier *in silico* study identified only six of eight canonical subunits in trypanosomatids [12], suggesting that either the two subunits Sec5 and Exo84, are genuinely missing, or too divergent to identify.

**Fig 1:**
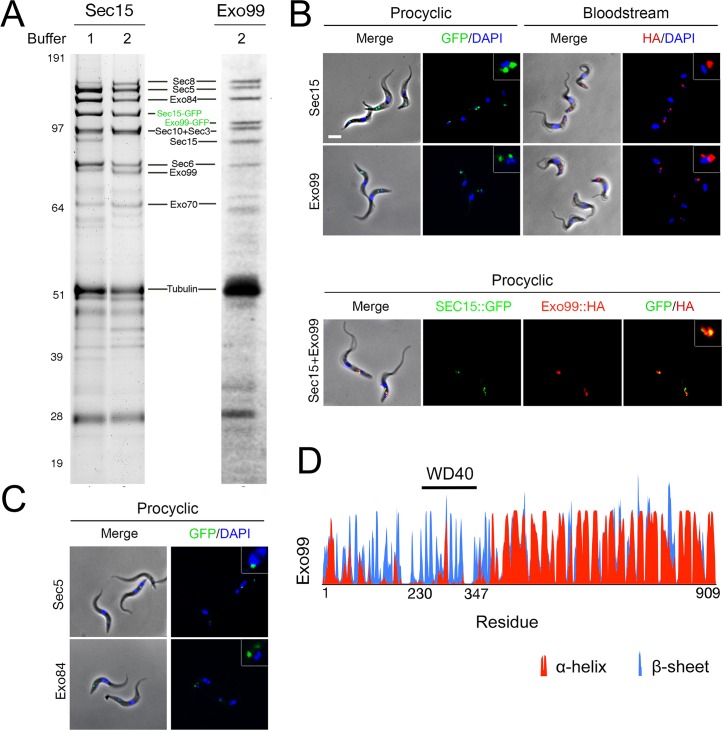
Identification of nine exocyst subunits in trypanosomes. (A) Immunoisolation from PCF cells constitutively expressing Sec15::GFP or Exo99::GFP. Pullouts were performed with the following buffers; (1) 20mM HEPES pH 7.4, 500mM NaCl 5% Triton and protease inhibitors; (2) 20mM HEPES pH 7.4, 250mM NaCitrate 5% CHAPS and protease inhibitors. (B) Immunofluorescence microscopy of PCF cells expressing Sec15::GFP and Exo99::GFP (green), BSF cells expressing Sec15::HA and Exo99::HA (red) and PCF cells co-expressing Sec15::GFP and Exo99::HA. DAPI was used to visualise DNA (blue). Scale bar, 5 μm. In both lifecycle stages Sec15 and Exo99 localise to the region between the nucleus and kinetoplast where the organelles of the endocytic and secretory pathways are found. (C) Immunofluorescence of PCF cells expressing Sec5::GFP and Exo84::GFP (green). Sec5 and Exo99 localise to the same region which indicates that they are part of the same complex. (D) Predicted secondary structure of Exo99 according to PSIPRED. The horizontal black line represents the polypeptide span with the N-terminus to the left, the y-axis indicates the confidence score of predicted secondary structure. Predicted α-helix are shown in red, predicted β-sheet in blue.

We purified the exocyst from *T*. *brucei* by affinity isolation and cryomilling (see methods) using a genomically-tagged TbSec15::GFP fusion protein as an affinity handle [42]. Initially, immunoprecipitations (IPs) were performed using various buffers and analysed by SDS-PAGE. Ultimately IPs using two comparatively stringent conditions, 20mM HEPES pH 7.4, 500mM NaCl, 0.5% (w/v) Triton X-100 and 20mM HEPES pH 7.4, 250mM NaCitrate, 0.5% (w/v) CHAPS were analysed by LCMS^2^ or MALDI-ToF/MS. From these purifications, orthologs to all eight mammalian and fungal exocyst subunits were identified, including Sec5 (Tb927.7.4490) and Exo84 (Tb927.7.5150). Trypanosome Sec5 and Exo84 are significantly larger than their animal or fungal orthologs, and have very limited regions of homology and low BLAST scores, explaining the failure to identify these subunits earlier. Based on Coomassie staining all subunits appear to be present at similar levels and predicted molecular weights were consistent with the electrophoretic migration positions for all subunits (Fig 1A). Further, only Sec15::GFP, and not untagged Sec15, was recovered, suggesting isolation of monomeric exocyst complexes.

### Exo99, a novel component of the trypanosome exocyst

We also identified a 99 kDa protein containing a predicted central WD40 domain encoded by Tb927.10.420. This protein co-purified with similar abundance to the canonical exocyst subunits; therefore we designated it as Exo99. To confirm Exo99 as an exocyst component, we co-purified the entire nonameric exocyst using Exo99::GFP as the affinity handle, which was strong evidence for stable association and assignment as a *bona fide* component (Fig 1A). Seeking other potential exocyst interacting proteins, Sec15::GFP and Exo99::GFP IPs were analysed by LCMS^2^, with or without dynamic exclusion, to maximise depth and provide semi-quantitative data on complex composition. Significantly, without dynamic exclusion all exocyst subunits, including Exo99, were recovered as top scoring identifications alongside frequent contaminants and two hypothetical proteins, Tb927.8.5580 and Tb927.11.11010 (formerly Tb11.01.2800) (S1 Table), that were later tested for exocyst interaction (see below). The architecture of Exo99 demonstrates both similarities and divergences from the canonical exocyst subunits: specifically retaining a significant degree of α-helical structure, but also carrying an additional predicted complete β-propeller domain, and which may suggest roles in novel protein-protein interactions.

### Exo99 co-localises with the exocyst at the flagellar pocket region

To further verify Exo99 as an exocyst subunit, we epitope-tagged Sec15 and Exo99 and examined their localisation by immunofluorescence (IF) in both lifecycle stages. Sec15::GFP specifically localised to the flagellar pocket region, the sole site of exocytosis in *T*. *brucei*, and at high resolution appears to encompass much of the pocket membrane (S1 Movie); [36]. Indeed, both Sec15 and Exo99 localise to one or two discrete puncta by conventional IF juxtaposed to the kinetoplast (the mitochondrial DNA of the parasite) in both procyclic (PCF) and bloodstream form (BSF) cells and with a distinct epitope tag (Fig 1B). Tagging Sec15 and Exo99 in PCF cells showed overlap between Sec15::GFP and Exo99::HA, further suggesting that these proteins are components of the same complex, and that it is unlikely that the tag influences targeting (Fig 1B). Additional fluorescence signal in regions distal to the kinetoplast may reflect the presence of exocyst components elsewhere, where they may participate in assembly and loading onto transport vesicles, as reported for mammalian cells [43,44]. Further, the locations of Sec5 and Exo84 as analysed by epitope-tagging and IF were indistinguishable from Sec15 or Exo99, providing additional evidence that all are most likely *bona fide* trypanosome exocyst components (Fig 1C).

We also assessed if the products of Tb927.8.5580 and Tb927.11.11010, identified in the Exo99 pullout, are potential exocyst partners, by determining their locations. Tb927.8.5580 encodes a 526kDa protein orthologous to Vps13, and localises to the membrane-trafficking active region between the kinetoplast and nucleus and is therefore a candidate interaction partner for Exo99 (S1 Fig). Vps13 is highly conserved across the eukaryotic lineage and plays a role in late or post-Golgi trafficking [45]. By contrast, Tb927.11.11010 did not co-localise with Exo99 and is therefore unlikely to be an exocyst component or binding partner (S1 Fig). Collectively, these data suggest that in trypanosomatids the exocyst is a nonameric complex with a possible interaction with the Golgi complex *via* Vps13. Whilst previous data indicates an interaction between trypanosome Sec15 and Rab11, no evidence for interactions with additional small GTPases was obtained here [36].

### Sec15 and Exo99 are essential

**Fig 2:**
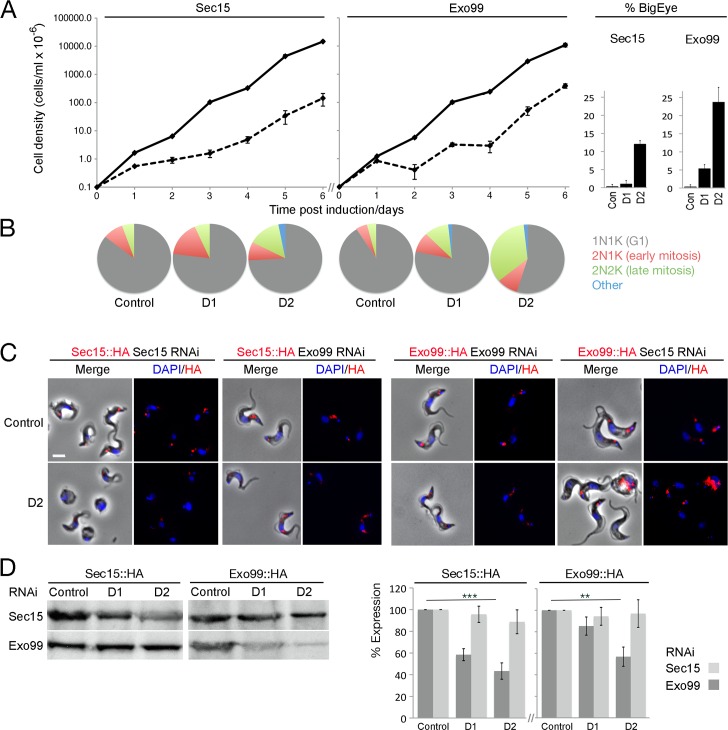
The exocyst is required for cell cycle progression in trypanosomes. (A) Cumulative growth curves of uninduced (solid line) and induced (dashed line) Sec15 and Exo99::RNAi lines. Sec15 and Exo99 depletion leads to a severe proliferative defect, further ~12% and ∼25% of Sec15 and Exo99::RNAi cells respectively have an enlarged flagellar pocket by 48h post induction. (B) Cell cycle analysis of Sec15 and Exo99::RNAi cells. For Exo99::RNAi cells a modest disruption in cell cycle progression manifested as an accumulation of 2K2N late-mitotic cells within 24 (D1) and 48h (D2) post induction; this defect is more severe in Sec15::RNAi cells. Representative result based on analysis of >100 cells. (C) Immunofluorescence microscopy of Sec15 and Exo99 depletion in BSF Sec15::HA and Exo99::HA cell lines. Sec15::RNAi cells show a ∼50% decrease of Sec15::HA signal (red), while the relative expression level of Exo99 remains unaltered, and *vice versa*. DAPI used to visualise DNA (blue). Scale bar, 5 μm. N = 50 G1 cells. (D) Western blot of Sec15 and Exo99::RNAi in BSF Sec15::HA and Exo99::HA cell lines. Sec15::RNAi cells have a ∼50% decrease to Sec15::HA 48h post induction, whilst expression of Exo99 remains stable, and *vice versa*. N = 3 independent experiments normalized to tubulin. Control: SMB cells uninduced; D1: 24 hr post induction; 48 hr post induction.

We used RNAi to ablate either Sec15 or Exo99 and to investigate possible roles for the exocyst in trypanosomes. Levels of both proteins were reduced by >50% within two days of RNAi induction, and a clear proliferation defect was observed (Fig 2). 12% and 25% of Sec15 and Exo99 RNAi cells respectively exhibited an enlarged flagellar pocket or ‘Big Eye’ (Fig 2A). This morphology, first described from clathrin knockdown, is characteristically induced by blocked endocytosis and/or a disruption in trafficking homeostasis [26].

We quantified the frequency of cells at various points along the cell cycle by staining both induced and uninduced cells with 4-,6-diamidino-2-phenylindole (DAPI) to track DNA-containing organelles. We observed in the Sec15::RNAi line a significant increase in the proportion of cells in early mitosis with two kinetoplasts and one nucleus (2K1N) and cells in late mitosis with two kinetoplasts and two nuclei (2K2N) (Fig 2B). A similar but more pronounced phenotype was observed in the Exo99::RNA line. These findings most likely reflect a defect in cytokinesis whilst the FP enlargement is a common phenotype associated with intracellular transport defects. The similarity of the phenotypes for Sec15 and Exo99 knockdowns supports the hypothesis that both are essential exocyst subunits.

*In trans* effects on complex stability can result from subunit depletion; for example, silencing one subunit in the trypanosome AP-1 complex leads to decreased expression of the other protein subunits [26]. However, silencing Sec15 did not affect localisation or expression level of Exo99 and *vice versa* (Fig 2C). Sec15 RNAi cells showed a ∼50% decrease of Sec15::HA signal but the relative level of Exo99::HA remained stable. Conversely, Exo99 RNAi cells showed a ∼50% decrease in Exo99::HA but the Sec15::HA expression level was unaltered (Fig 2C and 2D). These data suggest that, despite loss of function obtained from knockdown of individual subunits, the phenotype obtained is not due to overall decrease in complex abundance but rather appears as a result of loss of the individual subunits.

**Fig 3:**
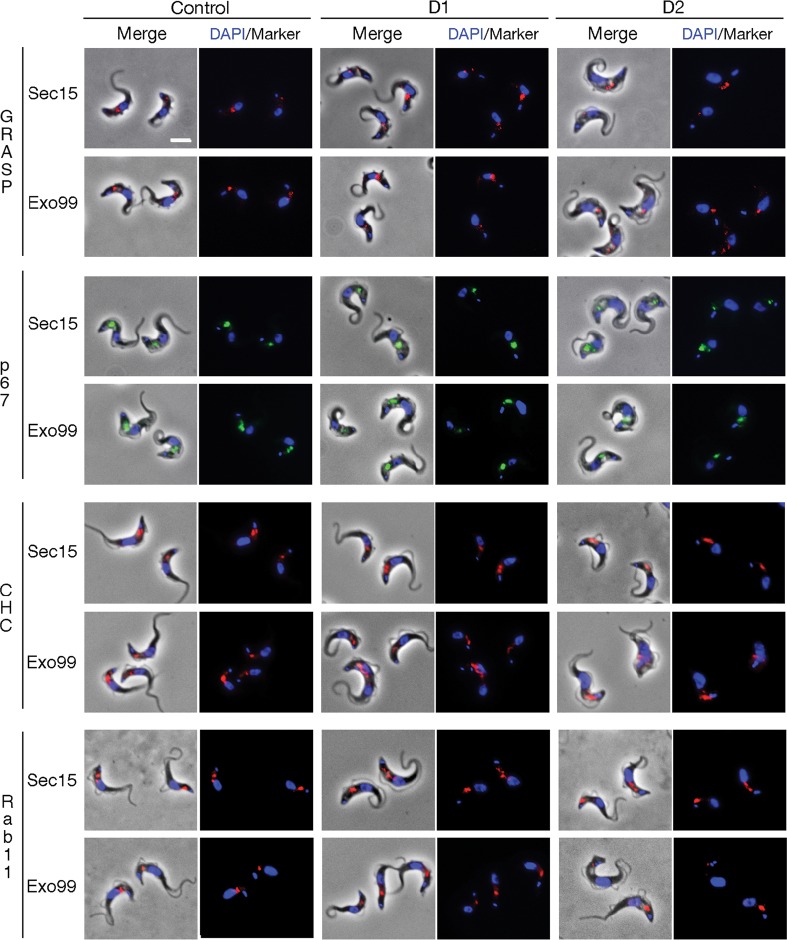
The exocyst is not required for endomembrane system maintenance or morphology. Immunofluorescence microscopy analysis of selected organelle markers or trafficking pathways for Sec15::RNAi and Exo99::RNAi cells at 24 (D1) and 48h (D2) post induction. Cells were stained with specific antibodies against GRASP (red), p67 (green), clathrin heavy chain (red) or Rab11 (red) and counter-stained with DAPI to visualise DNA (blue). The morphology and protein expression levels of all molecular markers remained stable. Scale bar, 5 μm.

Given the proliferative defect encountered from Sec15 or Exo99 silencing, we probed for a role in maintaining endomembrane compartments and distributions of clathrin and the Sec15 interactor Rab11. No impact on the locations of Golgi or lysosomal markers (GRASP and p67 respectively) was observed, at least in G_1_ (1K1N) cells that retained normal cellular morphology (Fig 3). Neither were there perturbations to localisation or expression levels of clathrin or Rab11, indicating that these proteins are not dependent on the exocyst for targeting (Fig 3). Some proteins associated with flagellar function were identified by mass spectrometry analysis of Exo99 pullouts e.g. PFR1, PFR2, BILBO1. Therefore we asked if suppression of Sec15 and Exo99 affected flagellum integrity by monitoring overall morphology and expression levels of the flagellar marker PFR2. No detectable alterations in the flagellum of RNAi mutants were observed, suggesting that the exocyst is not intimately involved with this organelle (S1 Fig).

### The exocyst has a role in endocytosis

**Fig 4:**
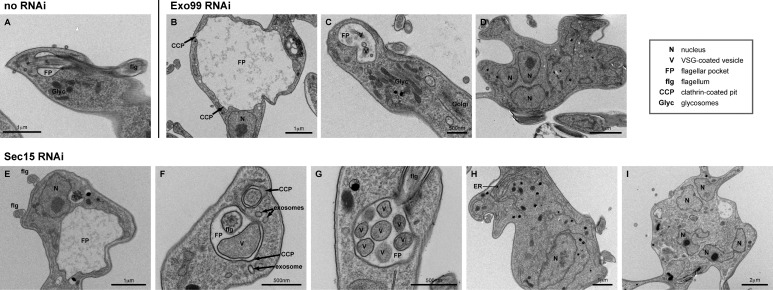
The exocyst is required for normal membrane trafficking. Representative transmission electron micrographs showing the effect of exocist subunit ablation 48h post-RNAi induction in BSF cells. The steady-state flagellar pocket has a small overall volume (A); ablation of Exo99 and Sec15 causes pocket enlargement (B, E), over-production of large VSG-coated vesicles inside the flagellar pocket (C, F, G), failure in cytokinesis (as illustrated by multiple nuclei in panels D and I) and ER hypertrophy (H). Despite large flagellar pocket volumes, indicative of endocytosis defect, clathrin is recruited to the surface membrane and able to assemble into coated pits and lattices (B, F).

Electron microscopy of RNAi mutants confirmed the ‘Big Eye’ effect upon silencing of Sec15 or Exo99 (Fig 4). Enlarged flagellar pockets contained sizable variant surface glycoprotein (VSG)-coated vesicles and also clathrin-coated membrane surrounding exocytic/recycling vesicles (Fig 4), suggesting that trafficking to and from the flagellar pocket is not completely abolished.

**Fig 5:**
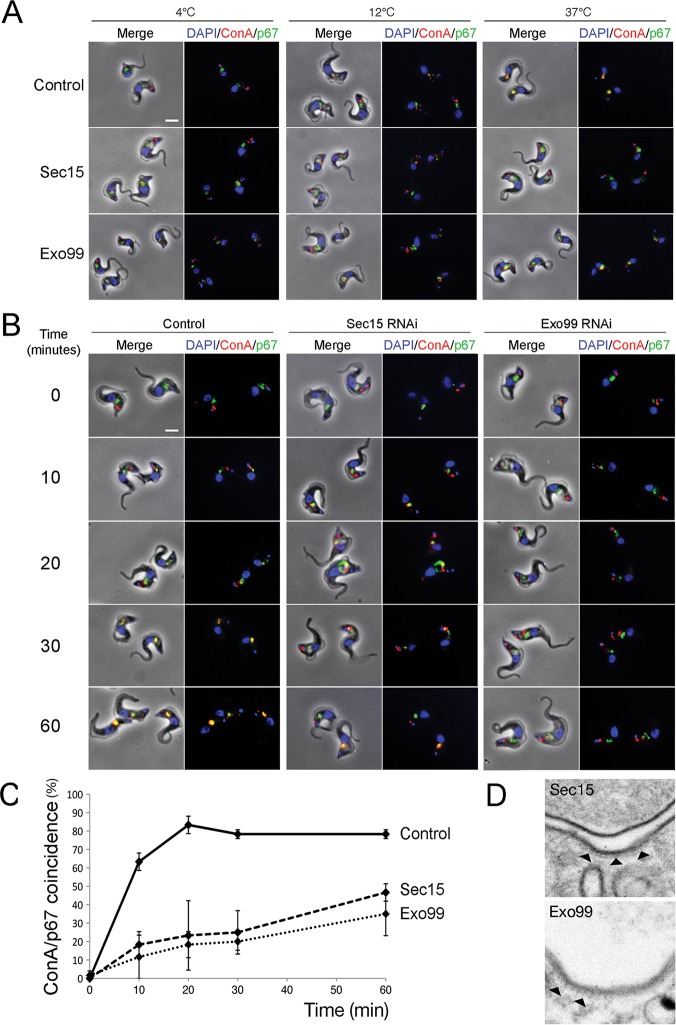
The exocyst is required for endocytosis. (A) Uninduced and induced Sec15 and Exo99::RNAi cells were allowed to accumulate FITC-conjugated ConA at the indicated temperatures. Fixed cells counterstained with DAPI (blue) and anti-p67 antibody for the lysosome (green). Both Sec15 and Exo99::RNAi lines show a defect in delivery of ConA to the lysosome: at 12°C and 37°C ConA remains predominantly localised at the flagellar pocket. (B) Dynamics of ConA uptake at 37°C 48h post RNAi induction. In control cells, ConA accumulates in a p67-positive compartment within 30 min. By contrast Sec15 and Exo99::RNAi cells exhibit a marked delay in delivery of ConA to the lysosome. In all images DAPI was used to visualise DNA (blue). Scale bar, 5 μm. (C) Quantitation of ConA lysosomal delivery at 37°C 48h post induction. N = 25 cells per time point per duplicate experiment. (D) Representative electron micrograph of curved flagellar pocket membrane coated by clathrin. Arrowheads point at clathrin coat.

Clathrin was also seen associated to the flagellar pocket membrane itself, of both Sec15 and Exo99 RNAi mutants, as slightly curved coated pit or flat coated lattices (Fig 4B, 4F and 4G; Fig 5D). This demonstrates that the impaired endocytosis in these mutants may not necessarily be caused by a failure in recruiting clathrin triskelions to the pocket membrane, or in assembling a functional clathrin coat around pits and lattices, but in proceeding to highly-curved pits and vesicle budding. In that regard, we have previously reported on a similar, post-recruitment phenotype upon ablation of clathrin interactors and adaptors [4,5], whereby a reduction in clathrin-coated pit density due to enlargement of the flagellar pocket volume was observed.

Organelles of the endocytic pathway appeared normal (Fig 3), although in Sec15-depleted cells ~15% showed an enlarged ER (Fig 4H). Ultrastructural analysis also confirmed the defect in cytokinesis, since the proportion of cells with multiple nuclei was significantly increased (Fig 4D and 4I). The presence of vesicles within the FP lumen is most likely a specific stress response, being a frequent observation in cells where trafficking is defective, but may also represent induction of an exosome pathway [46].

Since the appearance of the ‘Big Eye’ phenotype is characteristic of an endocytic defect, we studied the dynamics of endocytosis in Sec15 and Exo99 knockdown cells. VSG is the dominant mannose-containing protein at the trypanosome surface and binds the lectin Concanavalin A (ConA), which essentially reports on uptake of >90% of surface proteins. Cells were incubated with ConA at temperatures that allow internalisation into distinct endocytic compartments [47]: at 4°C ConA localises at the flagellar pocket; at 12°C ConA locates to both the flagellar pocket and early endosomes; while at 37°C ConA reaches the lysosome (here co-stained with anti-p67).

Cells depleted of Sec15 or Exo99 showed one discrete spot at the flagellar pocket at 4°C, consistent with cold block of endocytosis. However, at 12°C and at 37°C, ConA remained mostly localised at the flagellar pocket, indicating delays in trafficking events from the cell surface to the lysosome (Fig 5). Analysis of the dynamics of ConA uptake confirmed that both knockdown lines had substantial delays in all stages of endocytosis, from uptake at the flagellar pocket to lysosomal delivery. After 30 min, ∼80% of control cells showed greater than 50% signal overlap of ConA and p67, whereas only ∼20% of Sec15 and Exo99 RNAi cells displayed such signal overlap (Fig 5). Collectively, these results imply that knockdown of Sec15 or Exo99 generates a significant endocytic and lysosomal delivery defect without affecting lysosomal morphology. Interestingly, electron microscopy demonstrated clathrin-coated pits at the surface membrane and, together with unaltered clathrin distribution in Sec15 and Exo99 RNAi cells, suggests that the endocytic delay is not the result of failure to recruit clathrin to the flagellar pocket membrane, but that the flux through this step is impacted. We cannot, however, exclude the possibility that some of the decreased endocytic flux is a result of the generation of intralumenal vesicles. But given that the entire cell surface of trypanosomes turns over in ~7 minutes [48], this is likely a minor contributory factor.

### A role for the exocyst in maintaining cell surface components

**Fig 6:**
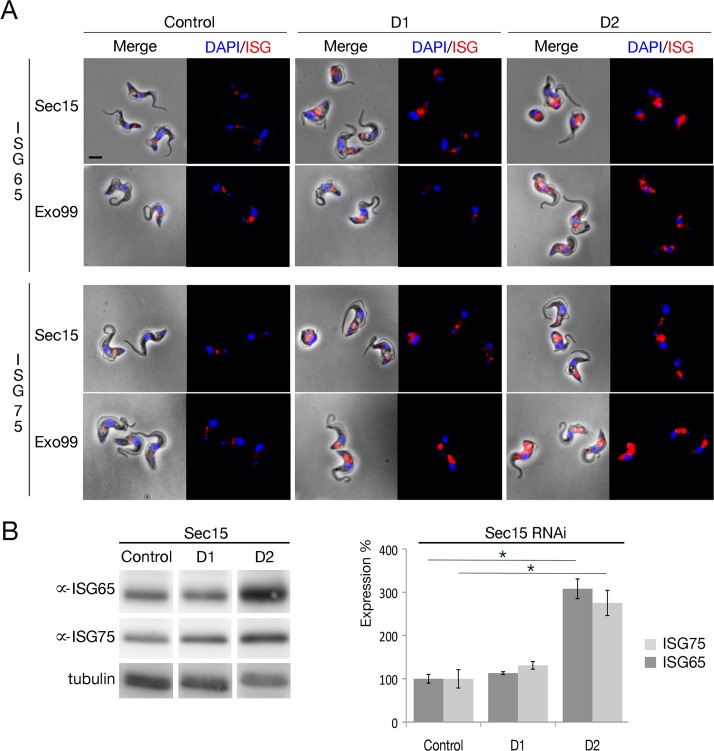
Exocyst mediates control of invariant surface glycoprotein expression levels. (A) The localisation and relative expression level of the intracellular ISG65 and ISG75 pool were assessed by immunofluorescence microscopy with specific antibodies (red), in control cells and in Sec15 and Exo99 RNAi cells at 24 and 48h post induction. In both RNAi lines the intracellular ISG pool is significant increased. Scale bar, 5 μm (B) Expression of total ISG65 and ISG75 in Sec15 RNAi cells at 24 (D1) and 48h (D2) post induction detected by Western blot. Steady-state ISG expression is significantly increased in Sec15 RNAi cells compared to control. N = 3 independent experiments normalized to tubulin signal.

As a further probe of trafficking, we analysed the impact of Sec15 and Exo99 RNAi on the major surface *trans*-membrane domain glycoproteins ISG65 and ISG75. These antigens have distinct trafficking itineraries, both from each other and VSG. However both proteins require ubiquitylation for turnover and endosomal targeting, and are sensitive to a number of endocytosis and recycling blockades [49–51]. Both Exo99 and Sec15 knockdown lines had significantly increased internal ISG pools (Fig 6), suggesting defects in late, pre-lysosomal events and/or recycling pathways [50]. Furthermore, ISG65 and ISG75 steady-state levels as assessed by Western blot were increased by ∼30% following Sec15 silencing (Fig 6), consistent with defective endocytic trafficking.

To provide an orthogonal, unbiased analysis of the surface proteome, we performed quantitative proteomics on whole cell lysates. A Sec15 RNAi line was generated using pRPa^SLi^ cells to increase RNAi penetrance and decrease clonal variability [52]. On induction the pRPa^SLi^ Sec15 RNAi line had an indistinguishable phenotype to the p2T7 Sec15 RNAi line described above, *albeit* with a more rapid onset. Up to 80% Big Eye cells were observed at 36hr post induction. Induced and uninduced pRPa^SLi^ Sec15::RNAi cells were grown in the presence of either light L-arginine and L-lysine (HMI11-R0K0), or L-arginine U-^13^C_6_ and L-lysine 4,4,5,5-^2^H_4_ (HMI11-R6K4) for 24 and 36h, as described in [51]. Whole cell lysates were resolved by 1D SDS-PAGE and subjected to proteomic analysis. Across all replicates we detected >1700 unique protein groups, amounting to ~25% of the predicted trypanosome proteome (S1 Table).

We considered significant only those proteins where changes were seen at both time points and where the change increased in a time-dependent manner. The impact of the Sec15 knockdown on ISG65 and ISG75 levels was in good agreement with Western blot analysis; Sec15 RNAi increased ISG75 abundance by ∼45–70%. For ISG65, four paralogues were increased by ∼50%. Interestingly, ISG64, another member of the ISG protein superfamily, also increased in abundance by ~65% together with three proteins with more distant ISG homology by 50–90%. Overall this quantitative analysis suggests a similar and significant impact on ISG and ISG-like protein families, potentially reflecting protection from degradation [51].

**Fig 7:**
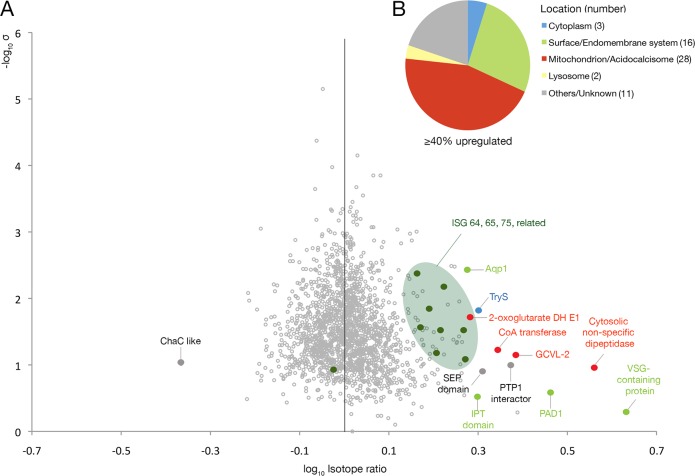
Quantitative proteomics by SILAC identifies altered surface protein expression mediated by exocyst knockdown. (A) Volcano plot of protein abundance changes at 36h post Sec15 RNAi induction. -log_10_ transformed SILAC ratios are plotted against -log_10_ transformed standard deviation. Data points representing protein groups significantly altered after 36 hours are labeled; ISG65 and ISG75 paralogs in dark green, other potential surface proteins in light green, mitochondrial proteins in red, cytoplasmic proteins in blue, lysosomal proteins in yellow, others in grey. AQP1: Aquaporin1, TryS: trypanothione synthetase; PTP1 interactor: Protein-tyrosine-phosphatase1-interacting protein; PAD1: Protein associated with differentiation 1; CoA transferase: Succinyl-coA:3-ketoacid-coenzyme A transferase; GCVL-2: Dihydrolipoyl dehydrogenase. (B) Predicted cellular localisation of proteins upregulated in Sec15 RNAi cells 36h post induction as reported by GO terms or as predicted by TMHMM2 for *trans-*membrane domains and PredGPI for GPI-anchor addition C-terminal signal sequences [89, 90]. Numbers of proteins in each category are in parenthesis.

In addition to ISGs, several other surface proteins had increased abundance (Fig 7), including a VSG-domain containing protein (Tb927.7.180), GRESAG5 (Tb927.7.6860), aquaglyceroporin1 (Tb927.6.1520), PAD1 and a predicted surface receptor from the plexin superfamily (Tb927.10.11140). Increased abundance of several additional proteins, predicted to function in mitochondrial pathways (especially the Krebs’ cycle) or key players of differentiation from bloodstream to procyclic form (PAD1 and a PTP1-interacting protein) [53, 54, 55] suggests that a differentiation switch was being activated or partially activated in these Sec15-depleted cells [56,57]. By contrast, no key endocytosis or cytoskeletal components were significantly altered, suggesting that the endocytosis block is not a direct result of decreased abundance of endocytic protein components.

Few proteins were significantly decreased by Sec15 depletion. Tb927.7.3220, a ChaC-like protein (member of the γ-glutamyl cyclotransferase-like superfamily) was decreased ~55% at 36hr post RNAi induction. Trypanothione synthetase (TryS), on the other hand, was increased ~25% at 24h and ~100% at 36h post induction. Altered expression levels of both proteins most likely represent defence against oxidant damage and may also be part of the cell differentiation process affected by Sec15 knockdown [58].

### The exocyst functions in endocytosis in metazoan cells

**Fig 8:**
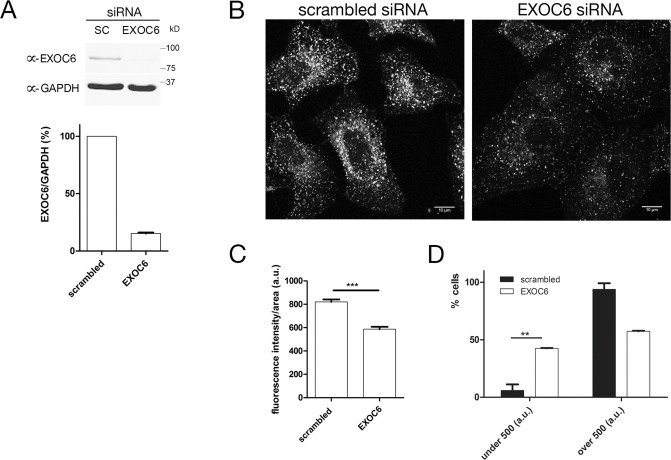
The exocyst functions in endocytosis in human cells. (A) Immunoblots of lysates prepared from HeLa cells treated with either scrambled or EXOC6 siRNA smart pools as shown in panel B, probed with anti-EXOC6 or anti-GAPDH as indicated. Knockdown levels of EXOC6 were quantified from 3 experiments of this type and are presented as a ratio of EXOC6/GAPDH signals. (B) HeLa cells depicting transferrin uptake. Cells treated with either scrambled or EXOC6 siRNA were imaged after the uptake of labelled transferrin (10 min). Representative fields of cells from 3 independent replicates. (C) The fluorescence intensity of >100 cells for each condition was determined using ImageJ and the data compared using an unpaired t test. *** P value < 0.0001. (D) The image intensity of >100 cells of each condition were binned into two groups, those with a signal intensity >500 arbitrary units, and those <500. Knockdown of EXOC6 was found to significantly increase the fraction of cells with a signal intensity <500 (** P value <0.01), consistent with a decrease in transferrin endocytosis.

While there is growing circumstantial evidence for roles for the exocyst in recycling and endocytosis in metazoan cells, this has not been directly measured. The extreme level of endocytosis in trypanosomes made this aspect of exocyst function quite obvious, so we asked if a similar function is present in mammalian cells. EXOC6, the human Sec15 ortholog, was depleted by EXOC6 siRNA smart pool transfection in HeLa cells, alongside transfection of scrambled siRNA as control. EXOC6 was depleted to ~15% at 48h post transfection (quantified as a ratio of EXOC6/GAPDH by Western blot; Fig 8A). Labelled transferrin uptake by HeLa was analysed at 48h post transfection with EXOC6 or scrambled siRNA by rendering cells quiescent for two hours in serum-free medium followed by incubation with 1ug/ml Alexa Fluor 488 conjugated transferrin for 10 minutes (Fig 8B). The fluorescence intensity of >100 cells for each sample showed a decrease in the transferrin signal to 25% in EXOC6 knockdown cells compared to scrambled siRNA (Fig 8C). The image intensity from each condition were binned into two cohorts, those with signal intensity >500 arbitrary units, and those <500. Knockdown of EXOC6 was found to significantly increase the fraction of cells with a signal intensity <500 by ~40%, consistent with a decrease in transferrin endocytosis (Fig 8D), confirming a role of exocyst in endocytotic events not only in the Kinetoplastida, but also in human cells. Although detailed kinetic analysis of this effect has not been undertaken here and we recognise that cell surface levels of transferrin receptor may be reduced (although total levels are unchanged (S2 Fig), these data strongly suggest a functional correlation of the effects observed in Kinetoplastida with effects in higher Eukarya [59].

## Discussion

Cellular complexity was revolutionised by the development of an endomembrane system during eukaryogenesis. The intervening period of over a billion years subsequently moulded these ancestral transport systems by both losses and gains to those now present in living cells [60]. Secondary loss of ancestral components is quite common,and likely a response to the requirements of individual organisms or lineages, and are reasonably easy to identify by comparative genomics [61]. By contrast, lineage-specific modifications, which are potentially very common, are difficult to identify *in silico*. The absence of extensive direct data in protozoa and many other taxa has precluded the full appreciation of how extensive a contribution lineage-specific innovations make to trafficking systems, and their biological consequences. We previously demonstrated many gains and losses in the endocytic system and surface proteome of trypanosomes [4, 5, 62, 63], which prompted analysis of the exocyst, originally identified as a component of late exocytic transport in budding yeast [17].

To address the question of composition and lineage-specific subunits, we purified the exocyst holocomplex. Replication of this isolation in *S*. *cerevisiae* [21] suggests that this is a generalisable purification strategy, but that surprisingly the trypanosome exocyst is nonameric, containing all canonical subunits plus Exo99. The earlier prediction of a simpler exocyst was likely due to extreme divergence for two subunits, but their direct identification provides robust evidence for their presence in the trypanosome exocyst, which likely suggests a significant level of mechanistic conservation. Indeed, whilst this work was in progress, Sec5 was predicted in trypanosomes using more sensitive modern algorithms [64]. However, the discovery of a ninth subunit, and one with a clear predicted protein interaction domain (i.e. an intact β-propeller) does suggest that additional and specific interactions may subtend the trypanosome exocyst and not the host complex. These may induce the Vps13 ortholog Tb927.8.5580 for example, which has not been suggested to interact with the yeast or mammalian exocyst and provides an additional connection to the Golgi complex.

Evidence that Exo99 is a *bona fide* exocyst subunit includes localisation, reverse immunoprecipitation and similarity of function indicated by knockdowns of Sec15 and Exo99. The presence of Exo99 also raises the possibility that the exocyst in other lineages contains non-canonical subunits, and the presence of an additional exocyst subunit further highlights evolutionary modifications in membrane trafficking pathways, underscoring the flexibility of these pathways across evolution [4, 5].

The exocyst displays extensive protein interactions but we did not detect stable interactions with GTPases or the cytoskeleton for example. For trypanosomes the GTPase complement at the plasma membrane is likely less complex than in animals and fungi as there is only a single Rho-like protein RHP [65], but such interactions were also not identified robustly in yeast for example [21]. We previously demonstrated an interaction between Sec15 and Rab11 in trypanosomes by yeast two hybrid, which likely reflects the difficulty in maintaining such connections biochemically [36]. In yeast, actin disruption leads to mistargeting of many exocyst subunits, suggesting that actin trafficking is needed for their targeting [66], but such evidence is not yet available for trypanosomes. Sec3 and Exo84 interact with WASH, suggesting that both WASH and exocyst control actin at endosomes in mammalian cancer cells [67]. Additional roles in cytokinesis in multiple organisms have also been described, for example a role for Exo70 in targeting Rab11 to the mammalian cleavage furrow [68], whilst in vascular plants there is an emerging role for Exo70 in an ER-to-vacuole pathway analogous to autophagy [69].

The connection with actin and the targeting of exocyst to endosomes via Rab11 has however suggested a role for exocyst in endocytic pathways, which was recently also supported by imaging of membrane trafficking in yeast [70]. An endocytic role for the trypanosome exocyst is supported by multiple lines of evidence: enlargement of the flagellar pocket, decreased turnover of multiple surface proteins and delayed delivery of endocytic probes to the lysosome. Significantly, both Sec15 and Exo99 generate this phenotype, suggesting that both subunits are required and, similarly to the canonical subunits, all nine are required by the trypanosome. Detection of an endocytic defect was aided by extreme flux in mammalian-infective trypanosome cells. The presence of vesicles within the FP lumen is further evidence for the importance of the exocyst in maintaining membrane trafficking and is potentially connected with generation of exosomes, as these vehicles are coated with VSG [46], but this remains speculative at present.

However, we could confirm an endocytic effect in mammalian cells using conventional transport assays, and suggest that regulation of endocytosis is likely an ancient, conserved exocyst function [59]. Hence, dual roles in transport to and from the cell surface, and integration with the cytoskeleton and multiple GTPases, confirms that exocyst is indeed a major hub controlling intracellular trafficking [25].

Exocyst subunit knockdown also provides new insight into the dynamics of the trypanosome cell surface. Two major cohorts of proteins, surface proteins/receptors and proteins involved in mitochondrial pathways, were found to increase in abundance upon ablation of Sec15. Whilst a VSG-related protein with unknown function and ISGs are clearly increased, the expression level of VSG itself was unaltered. This suggests that proteins that are rapidly transiting the recycling system are affected by exocyst function. This also indicates that several other ISG protein families are indeed expressed at the surface. Further, two components of the differentiation pathway from the bloodstream to the procyclic stage–the surface receptor PAD1 and an interactor of the cytosolic phosphatase PTP1 –were increased, suggesting a connection between exocyst-dependent trafficking pathways and bloodstream-to-procyclic differentiation. Significantly, this is consistent with an increase in abundance of a significant proportion of proteins involved in mitochondrial pathways, also a feature of this differentiation pathway [53, 54, 55, 56, 57].

In conclusion, we reveal new roles for the exocyst in endocytosis across eukaryotes and developmental pathways in trypanosomes, and demonstrate considerable evolutionary flexibility in retention of exocyst. The identification of Exo99 in trypanosomes also raises the possibility of additional, lineage-specific subunits in taxa that remain to be uncovered. Exo99 may provide opportunities for specific blockade to parasite exocytosis and endocytosis as this protein is present in the trypanosome and not the host.

## Methods

### Plasmid constructs for RNA interference and chromosome tagging

Primers for amplification of RNAi target fragments were designed using RNAit [71] to amplify a region unique to the gene of interest compared with the rest of the *T*. *brucei* genome. RNAi target fragments were PCR amplified using *Taq* DNA polymerase with gene-specific primers: Sec15for: AAGTCCCGTCATCGTTTCAC; Sec15rev: GCTCCCCTAGCCTTCTTTCGT; Exo99for: TGCGAAACGTTTGCTCATAG; Exo99rev: CATTCTCGCACTCCAACTGA and cloned into the tetracycline-inducible RNAi expression vector p2T7^TAblue^ [72], that was linearised with Eam1105I. Chromosome tagging with hemagglutinin (HA) or green fluorescent protein (GFP) was performed using the protocol and vectors described in [73] with the following gene-specific primers: Sec15for: CGGTTCCCCCTGAACCCGACGCAAATTGCGGATGATCTGTTAGCGTGGATCGCAAATAAGGAGGCCGCGCTAAAGCGAACTTTAGGTACCGGGCCCCCCCTCGAG; Sec15rev: TCCAACGTTGCAAGAGTGCACGCCGCCACCTTGAGAGCAACCATCCGCGATGTTCAGCCCATCAACCGTCAGACATGGATGTGGCGGCCGCTCTAGAACTAGTGGAT; Exo99for: TCGAAGAAAGGCGGTGACGAAATGTTAAATGAAATACTCGACACATGTGACGAGTTCTTTGACAGAGTTGCTGAAGATGCTGATGGTACCGGGCCCCCCCTCGAG; Exo99rev: CGGTAACATTCCCCTCACTGTGGGATTGGAACTGCCTTTGACGCCGAACACCTACGAAGGGCAAATATGAACTTTTAGAACTGGCGGCCGCTCTAGAACTAGTGGAT; Exo84for: CGAACAGGAGGTGACACCAGTAAAGGAGGTGTGACACTGTTTCACGTG TACAATATGTTGCTACACTACCTTGCGCCGCTAACAGGTACCGGGCCCCCCCTCGAG; Exo84rev: GCAACGGAAATATCTGCGTATGGCATTCCCCTGTATGGTATTTTTCAGTACCCCGTCAGAACCCTTCTATTCTCTTTCCGGTGGCGGCCGCTCTAGAACTAGTGGAT; Sec5for: ACTTTTTTGCCATCAGCTGCTGCCACTACCCCATTTGGTGGTGGTAAAACATCTACCCGTGCTTTCAAGAGATCAATACTACATGGTACCGGGCCCCCTGAG; Sec5rev: GGCACCTAAGGGACATCAGCGTAAAAGCAAACAAAAATAAACCAACAGGGCAAAACCAAAGCCACGAGCTGAAACAATCACTGGCGGCCGCTCTAGAACTAGTGGAT; Tb927.8.5580for: GGATGGGAATACGTTGGAAAAGAGAGTCACACCTCTACCGAGTTGAGGCGCCGTTGCTGGAAGCGAAGAATCCGAAAGGTGCTGGGTACCGGGCCCCCCCTCGAG; Tb927.8.5580rev: ACTATACGTCCCCCCCCCTTCGTACACTGCTTCAGCATCCAATAGACCCTCACCCGCTTAAACCAAAGCAACCAAAAACCATGGCGGCCGCTCTAGAATAGTGGAT; Tb927.11.11010for: GAGCAGCGGAAGCAATACCGTGAAGAGCTCATGAAGCAAATGCGCGAGAAATACGAATGGCAACTGAGCCACTTAGATGGTGTGGGTACCGGGCCCCCCCTCGAG; Tb927.11.11010rev: CTGGGACCACAACGTAAGGGGAACACGCGCGTGGGAGGCTTGGCCAG GCAGTACATCTGCATGTGCCCCTCCGGGCTCACTGGCGGCCGCTCTAG AACTAGTGGAT.

### Cell culture

Bloodstream form (BSF) *T*. *brucei brucei* MITat 1.2 (M221 strain) and procyclic form (PCF) *T*. *b*. *brucei* MITat 1.2 (Lister 427) were grown as previously described [74, 75]. Single marker BSF (SMB) cell lines were used for expression of tetracycline-inducible constructs. Expression of plasmid constructs was maintained using antibiotic selection at the following concentrations: G418 and hygromycin B at 1 μg/ml and phleomycin at 0.1 μg/ml for BSF, and G418 at 20 μg/ml and hygromycin B at 25 μg/ml for PCF.

### Transfection of *T. brucei*

Cells were harvested at 4°C, and for BSF the Amaxa nucleofector protocol [76] was followed using 10–25 μg of linearized DNA (either linearized plasmid for the RNAi lines, or ethanol-precipitated PCR product for chromosome-tagged lines). For PCF electroporation was performed with 5–15 μg of DNA using a Bio-Rad Gene Pulser II (1.5 kV and 25 μF).

### Comparative genomics of candidate exocyst components in eukaryotes

Candidate exocyst components were identified by scanning a panel of eukaryotic predicted proteomes with known exocyst component sequence queries using BLAST [77]. For each component, six queries were selected from the following species: *Homo sapiens*, *Saccharomyces cerevisiae*, *Trypanosoma brucei*, *Dictyostelium discoideum*, a chromalveolate (*Phytophthora capsici/Albugo laibachii/Phytophthora sojae/Phytophthora ramorum*), an Archaeplastida *(Arabidopsis thaliana/Chlamydomonas reinhardtii/Selaginella moellendorffii*). The top BLAST hits (an e value that gave greater than 100 hits was used as a selection threshold) from each of the six scans were pooled in a neighbour-joining tree after alignment with ClustalW [78]. Alignments were created using MUSCLE [79].

### Transfection of HeLa cells

HeLa cells (ATCC CCL-2^TM^, LGC, UK) were cultured as previously described [80]. For the knockdown cells were seeded out into 6-well plates onto 13mm glass coverslips and grown at 37°C until 50% confluent. siRNA Silencer Select EXOC6 or Silencer Select Negative Control #2 siRNA (Life technologies, UK) were transfected following the instructions for Dharmafect 4 (GE Healthcare, UK). Reduction in the extent of Tf accumulation were evident at 5 and 20 minutes of uptake (S2 Fig) in addition to the 10 minute time shown in Fig 8. Total cell transferrin receptor levels were not reduced by knockdown of EXOC6 (S2 Fig)

### Interactome analysis

Protein-protein interactions between Sec15/Exo99 and other trypanosome proteins were analyzed by immunoisolation after cryomilling of parasites. In brief 5x10^10^ procyclic cells habouring Sec15 or Exo99 genetically tagged at the C-terminus with GFP were lysed by mechanical milling in a Retsch Planetary Ball Mill PM200 using liquid nitrogen cooling (Retsch, United Kingdom). Aliquots of powder were thawed in the presence of solubilization buffer (20 mM 4-(2-hydroxyethyl)-1-piperazineethanesulfonic acid [HEPES], pH 7.4, 500 mM NaCl, 0.5% Triton or 20 mM HEPES, pH 7.4, 250 mM NaCitrate, 0.5% CHAPS) and Sec15::GFP/Exo99::GFP were isolated using llama anti-GFP antibodies coupled to Dynabeads. Affinity isolates were reduced in 50 mM DTT and alkylated using 75 mM iodoacetamide, fractionated by SDS-PAGE and analysed by mass spectrometry [81, 82]. Peptides generated by tryptic digest were identified using a SCIEX prOTOF 2000 matrix-assisted laser desorption/ionization orthogonal time-of-flight Proteomics Mass Spectrometer (PerkinElmer, Akron, OH) and peak lists were submitted to ProFound and searched against an in-house-curated *T*. *brucei* database using data from GeneDB (www.genedb.org). Alternatively liquid chromatography-tandem mass spectrometry was performed on a Velos Orbitrap (Thermo Electron, Waltham, MA) and proteins identified on the Global Proteome Machine database (www.thegpm.org) as described previously [82].

### Immunofluorescence microscopy

*T*. *brucei* cells were prepared as previously described [31]. Antibodies were used at the following concentrations: mouse anti-HA (Santa Cruz Biotechnology, Heidelberg, Germany) 1:1000; mouse anti-tubulin clone KMX-1 (Millipore, Watford, UK) 1:1000; mouse anti-p67 (from J. Bangs, University of Wisconsin-Madison) 1:1000; rabbit anti-GRASP (from Graham Warren, Vienna, Austria) 1:500; rabbit anti-clathrin 1:500; rabbit anti-Rab11 1:500; rabbit anti-PFR2 (L8C4 from Keith Gull, Oxford, United Kingdom) at 1:100; and rabbit anti- ISG65 and rabbit anti-ISG75 (from Mark Carrington, Cambridge, United Kingdom) 1:1000 in PBS. Widefield epifluorescence images were acquired using a Nikon Eclipse E600 epifluorescence microscope equipped with a Hamamatsu ORCA charge-coupled device camera, and data were captured using MetaMorph (Universal Imaging, Marlow, UK). Quantitation was performed on raw images gathered under nonsaturating conditions using ImageJ [83] software. HeLa cells on glass coverslips were immunostained as outlined in [84] and images acquired using a Zeiss LSM5 confocal microscope and Pascal software (release version 4), with a 63x oil immersion, 1.4 numerical aperture lens.

### Fast, isothermal fixation and electron microscopy

To minimise perturbations to endocytosis due to live cell handling, all cell lines analysed ultrastructurally were fixed in culture by the addition of isothermal glutaraldehyde to the culture flask to a final concentration of 2.5%, as previously described [85]. The culture flask was gently rocked for 10 min at 37°C, after which time fixed cells in medium were harvested by centrifugation at 800 g for 10 min and resuspended in buffered 2.5% glutaraldehyde for 30 min at room temperature. Fixed cells were post-fixed in 1% osmium tetroxide for 30 min at room temperature, en-bloc-stained with 1% aqueous uranyl acetate, dehydrated through acetone and embedded in epoxy resin. Ultra-thin sections (70 nm) were post-stained with 2% aqueous uranyl acetate and lead citrate. Electron micrographs were taken using a Tecnai G2 (FEI) operating at 80kV.

### Western immunoblotting

*T*. *brucei* whole cell lysate of 10^7^cells/lane was resolved by SDS-PAGE and transferred to polyvinylidene fluoride membranes (Millipore). Antigens were visualized using standard methods. Primary antibodies were used at the following concentrations: mouse anti-HA (Santa Cruz Biotechnology, Heidelberg, Germany) at 1:10000 and rabbit anti- ISG65 and rabbit anti-ISG75 (from Mark Carrington, Cambridge, United Kingdom) 1:10000 in TBST (Tris-buffered saline + 0.05% Tween 20); Primary antibody binding was detected using secondary anti-IgG horseradish peroxidase (HRP) conjugates (Sigma-Aldrich) at 1:10000. Detection of HRP-conjugated secondary antibodies was by chemiluminescence using luminol (Sigma-Aldrich) and X-ray film (Kodak).

HeLa cells were washed three times in ice-cold PBS on ice, then lysed in ice-cold lysis buffer (50mM Hepes pH 7.2, 100mM KCl, 5mM NaCl, 1mM MgCl2, 0.5mM EGTA, 1mM EDTA, 0.1% (v/v) Triton X100, 1 tablet cOmplete EDTA-free (Sigma-Aldrich, UK), 1mM DTT). The lysate was passed through a 26 gauge needle 10 times, rested on ice for 20 min and homogenisation repeated. Insoluble cell debris was sedimented at 16,000xg for 15 minutes at 4°C, and the supernatant denatured in Laemmli buffer for 5 minutes at 95°C. 20–40 ug solubilized protein were subjected to 10% SDS-PAGE and subsequently transferred onto Nitrocellulose membrane. Mouse anti–glyceraldehyde-3-phosphate dehydrogenase (Ambion, UK) was used 1:80,000 and exocyst component complex 6 (Sigma Aldrich, UK) was used 1:1,000, Secondary antibodies, LI-COR antibodies rabbit IRDye 680RD and mouse IRDye 800CW (LI-COR, Lincoln, USA) were diluted in Odyssey Blocking Buffer (LI-COR, Lincoln, USA) and used in a 1:10,000 dilution. The blots were visualised with a LI-COR Odyssey-SA and quantified using ImageJ.

### ConA uptake assay

The assay was described previously [31]. Briefly 1.5 x 10^6^ cells were harvested at 800 *g* for 10 min at 4°C, washed with serum-free HMI-9 medium supplemented with 1% bovine serum albumin (BSA), and incubated in serum-free HMI-9 medium with 1% BSA for 20 min at 4°C, 12°C, or 37°C. Fluorescein isothiocyanate (FITC)-conjugated concanavalin A (ConA) (Molecular Probes, 5 μg/ml) was added, and the cells were incubated for a further 30 min at the above temperatures to allow uptake. Cells were washed with PBS and harvested by centrifugation at 800 *g* for 10 min at 4°C, fixed, and counter-stained for immunofluorescence as described above. To check ConA uptake dynamics, the same procedure was followed, except that cells were harvested by centrifugation, fixed, and stained after 0, 10, 20, 30, and 60 min of ConA incubation at 37°C. Images were captured using METAMORPH software (Universal Imaging Corp.) Quantification of the areas of fluorescence was performed on raw images, from identical exposures under non-saturating conditions with the Metamorph Region Measurements function.

### Transferin uptake assay HeLa cells

Cells were rendered quiescent for 2h in serum-free medium, followed by addition of 1ug/ml Alexa Fluor 488 conjugated transferrin (Molecular Probes, UK) for 10 min. Cells were rapidly washed in ice-cold PBS and fixed as described above. The uptake of transferrin into the cells was measured using ImageJ to determine the level of fluorescence intensity in a drawn region. For each condition a total of 100–150 cells were measured and the integrated density was divided by area (intden/area).

### SILAC labeling

HMI11 for SILAC was prepared as described in [86]: IMDM depleted of L-Arginine and L-Lysine (Thermo) was supplemented with 10% dialyzed (10 kDa molecular weight cutoff) fetal bovine serum (Dundee Cell Products), 4 μg/ml folic acid, 110 μg/ml pyruvic acid, 39 μg/ml thymidine, 2.8 μg/ml bathocuproinedisulfonic acid, 182 μg/ml L-cysteine, 13.6 μg/ml hypoxanthine, 200 uM β-mercaptoethanol, 0.5 μg/ml Phleomycin and 2.5 μg/ml Hygromycin. Finally either normal L-Arginine and L-Lysine (HMI11-R0K0), or L-Arginine U−^13^C_6_ and L-Lysine 4,4,5,5-^2^H_4_ (HMI11-R6K4) (Cambridge Isotope Laboratories) were added at 120 μM and 240 μM respectively. RNAi was induced by addition of 1 μg/ml tetracycline and at the indicated times equal numbers of induced and uninduced cells were mixed, harvested by centrifugation, washed twice with PBS containing Complete mini-protease inhibitor cocktail (Roche) and resuspended in Laemmli sample buffer containing 1 mM dithiothreitol. Samples were generated in duplicate, sonicated and aliquots containing 5x10^6^ cells were separated on a NuPAGE bis-tris 4–12% gradient polyacrylamide gel (Invitrogen) under reducing conditions. The Coomassie-stained gel lane was excised in eight, destained, then subjected to tryptic digest and reductive alkylation. The eight fractions were subjected to LC-MS/MS and mass spectra analysed using MaxQuant version 1.5 [87] searching the *T*. *brucei* 927 annotated protein database (release 8.1) from TriTrypDB [88]. Minimum peptide length was set at six amino acids, isoleucine and leucine were considered indistinguishable and false discovery rates (FDR) of 0.01 were calculated at the levels of peptides, proteins and modification sites based on the number of hits against the reversed sequence database. SILAC ratios were calculated using only peptides that could be uniquely mapped to a given protein. If the identified peptide sequence set of one protein contained the peptide set of another protein, these two proteins were assigned to the same protein group.

## Supporting Information

S1 Fig (A) Tb927.8.5580, a Vps13 homolog in trypanosomes, is a candidate interactor of Exo99. Tb927.8.5580 and Tb927.11.11010, identified in Exo99 immunoisolations, were genomically-tagged with HA (red) in PCF cells. The intracellular location was determined by immunofluorescence, and cells were counterstained with DAPI for DNA (blue) Scale bar, 5 μm. Vps13 localises to the region between nucleus and kinetoplast supporting its possible interaction with Exo99. Tb927.11.11010, a hypothetical protein, shows a cytosolic localisation and is therefore not validated as an interaction partner. (B) Sec15 and Exo99 knockdown does not affect relative expression level of PFR2 or flagellar morphology. Immunofluorescence of fixed Sec15::RNAi and Exo99::RNAi cells 48h post induction with a specific antibody against PFR2 (green) shows no obvious alteration in either flagellum morphology or signal intensity. PFR2: paraflagellar rod protein 2.
(PDF)

S2 Fig Quantitation of Transferrin levels in EXO6 knockdowns. Panel A: Western blots for expression levels of transferrin receptor (TfR), GAPDH (as loading control) and EXO6 (exocyst target) monitored in whole HeLa cell extracts exposed to either scrambled (Scr) or EXOC6 siRNA smartpools. Panel B: HeLa cells treated with either scrambled (Scr) or EXOC6 siRNA smartpools were examined for fluorescent transferrin uptake after exposure of cells to fluorescent transferrin for the times indicated. The fluorescence intensity of 50–100 cells for each time point was determined using ImageJ and the average fluorescence intensity in EXOC6 knockdown cells expressed as a percentage of the corresponding value in the scrambled siRNA-treated group (Scr). Shown above is a typical experiment, and the values reported are mean ± s.d. Based on such data, a more extensive analysis of uptake was performed as outlined in Fig 8 at ten minutes as a representative time point.
(PSD)

S1 Table Sec15 and Exo99-interacting proteins and abundance of selected protein groups following knockdown against Sec15. Worksheets (A-C) The most significant 50 protein identifications from an LC-ESI/MS^2^ analysis from a Sec15::GFP immunoisolation (A) with and (B) without dynamic exclusion and (C) from a Exo99::GFP immunoisolation without dynamic exclusion. Protein IDs are ranked by the expectation value according to ProFound. Canonical exocyst subunits and Exo99 are highlighted in red, and other proteins studied further in this work are in blue. Worksheets D and E: SILAC analysis of whole cell proteome at 24 and 36 hours knockdown of Sec15. Values represent average percentage protein abundance relative to uninduced cells ± standard deviation. In several cases abundance for specific peptides falls below the detection limit—for protein groups that are part of a multigene family such as ISGs this frequently reflects assignment of specific spectra to a single accession number. Worksheet F: List of proteins significantly upregulated in Sec15::RNAi cells 24 and 36h post induction with biological function as assigned in GO:term and cellular localisation as assigned in GO:terms or predicted by SignalP [91] and TMHMM [89].
(XLSM)

S1 Movie In the flagellar pocket of bloodstream form trypanosomes endocytic and exocytic sites are in close proximity but do not overlap. OMX super-resolution image of fixed Sec15::GFP (red) bloodstream form cells. Clathrin was visualised with a specific antibody against the clathrin heavy chain (green) and DAPI was used to visualise DNA (blue), and shows the location of the nucleus (large structure) and kinetoplast (small structure). The kinetoplast is located very close to the flagellar pocket and connected via physical interaction to the flagellum.
(AVI)
