# Supplementary figures and images for: The Trypanosome Exocyst: A Conserved Structure Revealing a New Role in Endocytosis
Source: PLoS Pathog. 2017 Jan 23;13(1):e1006063. doi: 10.1371/journal.ppat.1006063 (PMC5256885; doi:10.1371/journal.ppat.1006063)

A

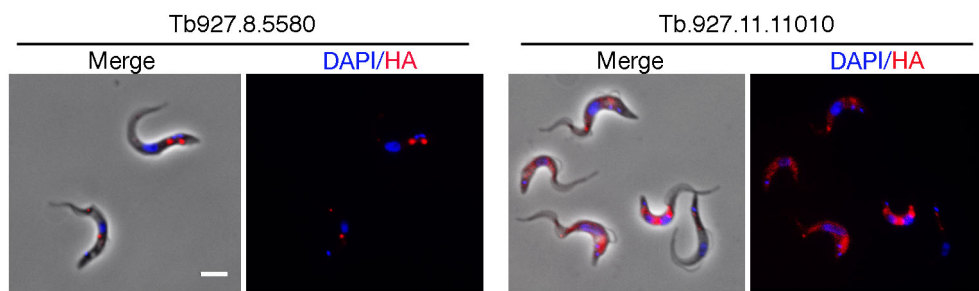

B

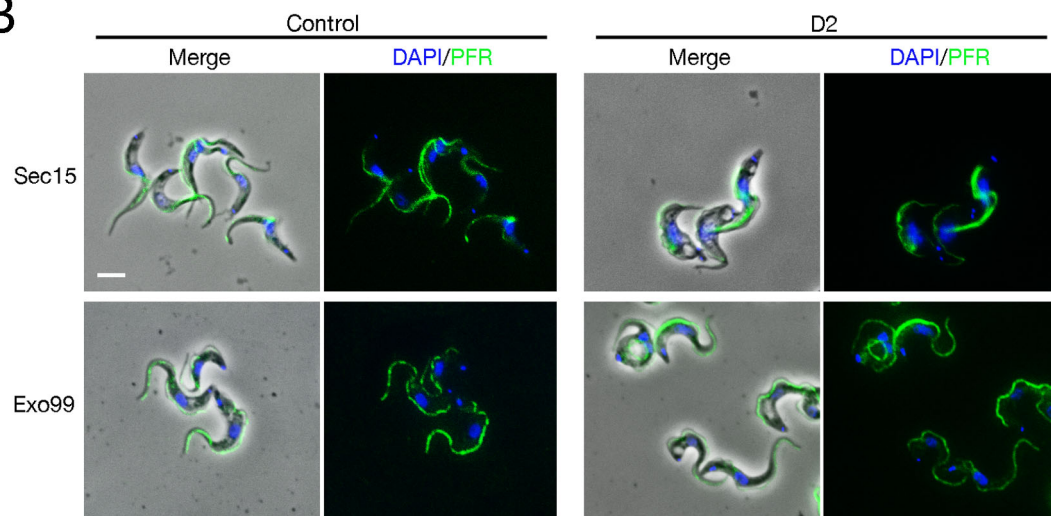

Supplement: S1 Fig — (A) Tb927.8.5580, a Vps13 homolog in trypanosomes, is a candidate interactor of Exo99. Tb927.8.5580 and Tb927.11.11010, identified in Exo99 immunoisolations, were genomically-tagged with HA (red) in PCF cells. The intracellular location was determined by immunofluorescence, and cells were counterstained with DAPI for DNA (blue) Scale bar, 5 μm. Vps13 localises to the region between nucleus and kinetoplast supporting its possible interaction with Exo99. Tb927.11.11010, a hypothetical protein, shows a cytosolic localisation and is therefore not validated as an interaction partner. (B) Sec15 and Exo99 knockdown does not affect relative expression level of PFR2 or flagellar morphology. Immunofluorescence of fixed Sec15::RNAi and Exo99::RNAi cells 48h post induction with a specific antibody against PFR2 (green) shows no obvious alteration in either flagellum morphology or signal intensity. PFR2: paraflagellar rod protein 2. (PDF) [file ppat.1006063.s001.pdf]
